# The transcription elongation factor TCEA3 induces apoptosis in rhabdomyosarcoma

**DOI:** 10.1038/s41419-020-2258-x

**Published:** 2020-01-27

**Authors:** Noor Kazim, Abhinav Adhikari, Teak Jung Oh, Judith Davie

**Affiliations:** 1 Department of Biomedical Science, Cornell University, Ithaca, NY 14850 USA; 2 Department of Biochemistry and Molecular Biology and Simmons Cancer Institute, Southern Illinois University School of Medicine, Carbondale, IL 62901 USA; 3 Department of Biochemistry, University of Illinois Urbana, Champaign, IL 61820 USA

**Keywords:** Paediatric cancer, Apoptosis

## Abstract

TCEA3 is one of three genes representing the transcription elongation factor TFIIS family in vertebrates. TCEA3 is upregulated during skeletal muscle differentiation and acts to promote muscle specific gene expression during myogenesis. Rhabdomyosarcoma (RMS) is a pediatric cancer derived from the muscle lineage, but the expression or function of TCEA3 in RMS was uncharacterized. We found that TCEA3 expression was strongly inhibited in RMS cell lines representing both ERMS and ARMS subtypes of RMS. TCEA3 expression correlates with DNA methylation and we show that TBX2 is also involved in the repression of TCEA3 in RMS cell lines. Ectopic expression of TCEA3 inhibited proliferation of RMS cell lines and initiated apoptosis through both the intrinsic and extrinsic pathways. We found that only pan-caspase inhibitors could block apoptosis in the presence of TCEA3. While expression of TCEA3 is highest in skeletal muscle, expression has been detected in other tissues as well, including breast, ovarian and prostate. We found that ectopic expression of TCEA3 also promotes apoptosis in HeLa, MCF7, MDA-231, and PC3 cell lines, representing cervical, breast, and prostate cancer, respectively. Restoration of TCEA3 expression in RMS cell lines enhanced sensitivity to chemotherapeutic drugs, including TRAIL. Thus, TCEA3 presents a novel target for therapeutic strategies to promote apoptosis and enhance sensitivity to current chemotherapeutic drugs.

## Introduction

Rhabdomyosarcoma (RMS) is a pediatric cancer of mesenchymal origin thought to arise from myogenic precursors in the skeletal muscle lineage^1^. RMS is classified into two major subtypes including the embryonal subtype (ERMS), which is the most common form of the disease and the alveolar subtype (ARMS), which is the more metastatic and aggressive subtype. ERMS is characterized by the loss of heterozygosity (LOH) at the 11p15 locus^2^ while ARMS is characterized by the chromosomal translocation t (2;13) (q35; q14) or t (1;13) (q36; q14), which generate chimeric PAX3-FOXO1 or PAX7-FOXO1 oncogenic fusion proteins^3,4^.

TCEA3 is one of three genes representing the transcription elongation factor TFIIS in vertebrates^5^. TCEA3 has been found to be highly expressed in embryonic stem cells, unlike the other family members TCEA1 or TCEA2, where it regulates the differentiation potential of the cells^6^. We have shown that TCEA3 is upregulated upon skeletal muscle differentiation and acts as a co-factor for the myogenic regulatory family (MRF)^7^. Recent studies have shown that TCEA3 expression is low in ovarian cancer cell lines compared to normal ovarian epithelial cells and ectopic expression of TCEA3 in ovarian cancer cell lines induces the caspase-dependent mitochondrial cell death pathway^8^. TCEA3 has also been shown to attenuate proliferation and induce apoptosis of gastric cancer cell lines^8^.

Apoptosis is a strictly controlled physiological process that triggers the cell to eliminate itself depending on signaling events in the dying cell^9^. It is controlled by a family of proteolytic enzymes known as the caspase (cysteine-dependent aspartate directed proteases) family that has the ability to activate each other^10,11^. Caspases are divided into two groups depending on their position in the apoptotic cascade; the upstream initiator caspases (caspase 9, 2, 8, and 10) and the downstream executioner caspases (caspase 3, 6, and 7)^12,13^. One of the hallmarks of cancer progression is apoptosis deficiency^14^ and promoting apoptosis in cancer cells has the potential to treat cancer^15^.

Apoptosis can be induced via two main mechanisms involving either the activation of death receptors on the cell surface (extrinsic pathway) or the mitochondria (intrinsic pathway)^16,17^. Extrinsic apoptosis is activated through stimulation of death receptors with death ligands such as tumor necrosis factor (TNF) that ultimately results in caspase activation and cell death^18,19^. Intrinsic apoptosis is triggered in response to intracellular signals that activate pro-apoptotic proteins that lead to mitochondrial outer membrane permeabilization and caspase activation. Bcl-2 family proteins are key regulators of this pathway.

In view of its putative role in differentiation and apoptosis during development, we sought to understand the expression and function of TCEA3 in RMS. We found that TCEA3 expression is low in RMS and show that both TBX2 and DNA methylation contribute to the repression of *TCEA3*. Surprisingly, we found that ectopic TCEA3 expression inhibited proliferation and initiated apoptosis in RMS cell lines representing both subtypes. Additionally, we show that TCEA3 can initiate apoptosis in cancer cell lines representing cervical, breast, and prostate cancer. TCEA3 promotes apoptosis through both the intrinsic and extrinsic pathways, and only pan-caspase inhibitors can block apoptosis in the presence of TCEA3.

## Results

### TCEA3 expression is downregulated in RMS cell lines compared with normal myoblast cell lines

**Fig. 1:**
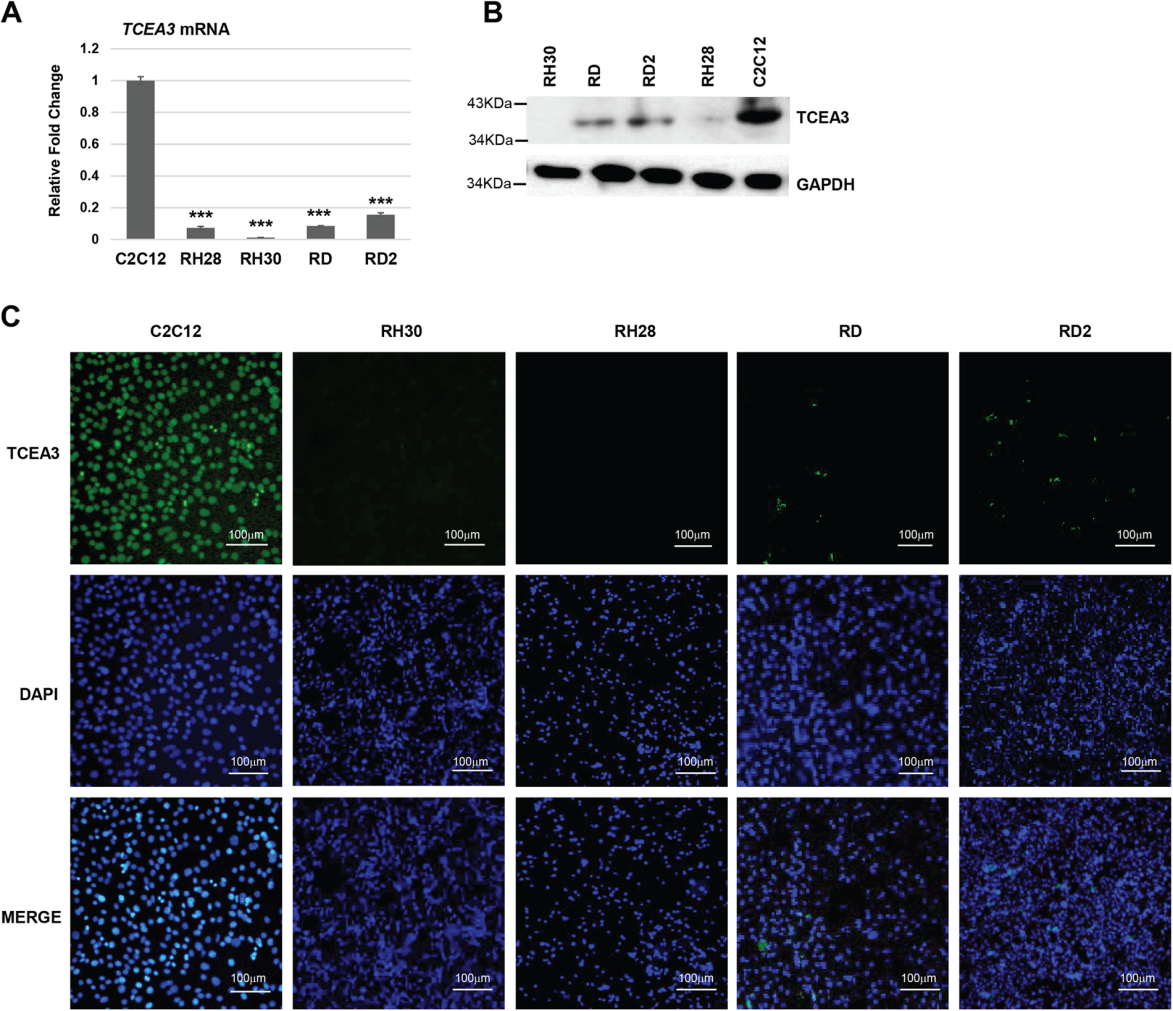
TCEA3 expression is downregulated in RMS. **a** TCEA3 mRNA expression was assayed in C2C12 cells, ARMS (RH28, RH30) and ERMS (RD, RD2) cell lines, respectively, by qRT-PCR. **b** Western blot with antibodies against TCEA3 to check expression in ERMS (RD, RD2), ARMS (RH28, RH30), and C2C12 cells. **c** TCEA3 immunostaining with antibodies against TCEA3 and DAPI was used to stain the nuclei. Scale bar is 100 µm. Error bars are S.E.M, Student's *t* test; ***p < 0.001 with respect to C2C12, *n* ≥ 3 biological replicates).

**Fig. 2:**
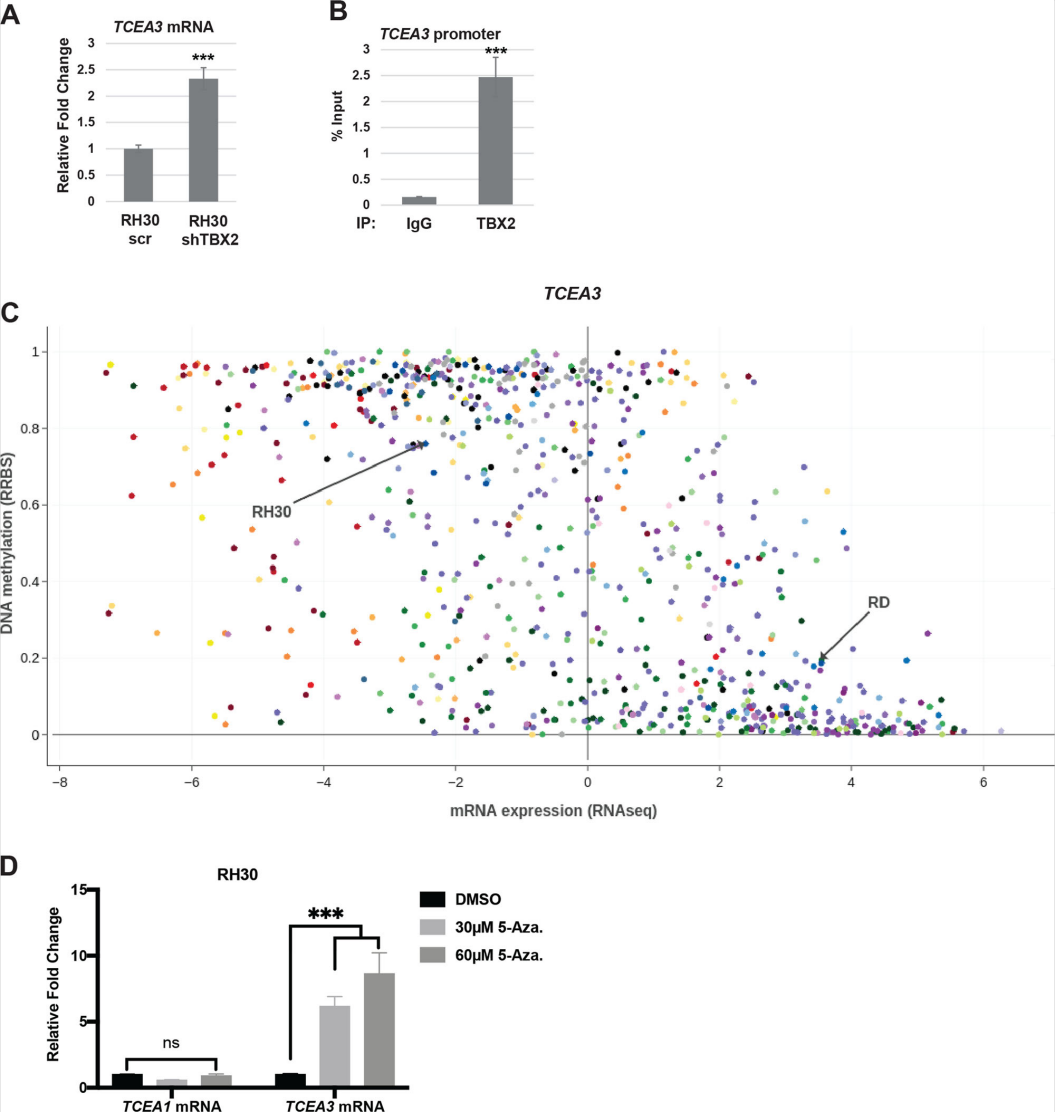
TCEA3 is repressed by TBX2 and expression correlates with DNA methylation. **a** RH30 cells depleted for TBX2 or scr control were assayed for mRNA expression of TCEA3 by qRT-PCR. **b** Chromatin immunoprecipitation (ChIP) assays with antibodies against TBX2 and IgG were assayed with primers against the *TCEA3* promoter. **c** Scatterplot for TCEA3 mRNA expression (RNAseq) and *TCEA3* locus methylation level (RRBS) was generated for 834 cancer cell lines using the Cancer Cell Line Encyclopedia (CCLE) database, and the plot was edited using Plotly tool. Each dot represents a cell line. mRNA expression value below 0 have no or negligible expression, and the DNA methylation levels values ranging from 0 to 1 denoting unmethylated to fully methylated locus, respectively. **d** Inhibition of DNA methylation derepresses TCEA3 expression in RH30 cells. mRNA expression of TCEA3 and TCEA1 were assayed in RH30 cells treated with either DMSO vehicle control or 5-aza-2’deoxycytidine DNA methyltransferase inhibitor at 30 μM and 60 μM concentrations for 48 h by qRT-PCR. Error bars are S.E.M. Student's *t* test; ns represents ‘not significant’, ***p < 0.001 with respect to control, *n* ≥ 3 biological replicates.

To understand the role of TCEA3 in RMS, we first characterized the expression of TCEA3 in four RMS cell lines representing both ERMS (RD and RD2) and ARMS (RH30 and RH28). Expression was compared with the C2C12 cell line, a murine cell line commonly used as a model for myogenesis. We found that TCEA3 expression was downregulated in both ERMS and ARMS cell lines at the level of mRNA (Fig. 1a), and protein (Fig. 1b) when compared to the level of TCEA3 in proliferating C2C12 cells, where TCEA3 expression is relatively low^7^. TCEA3 protein was almost undetectable in ARMS cell lines and weakly expressed in ERMS cell lines (Fig. 1b). As cancer cell populations are heterogeneous, we also examined TCEA3 expression by immunofluorescence to determine if TCEA3 was present in a subpopulation of RMS cells. We found that TCEA3 expression was uniformly low in both ARMS cell lines (Fig. 1c). In ERMS cell lines, faint TCEA3 staining that was more concentrated in a small subset of cells could be detected, indicating that TCEA3 expression may be heterogeneous in ERMS (Fig. 1c). We have recently shown that *TCEA3* is directly activated by myogenin (MYOG) in skeletal muscle^7^. The MRFs are thought to be inactive in RMS cells^20^, yet still required for RMS viability^21^. We have shown that TBX2 acts as a potent oncogene in RMS by repressing key genes required for cell cycle exit such as *CDKN1A* (P21), *CDKN2A* (P14), and *PTEN* and inhibiting the activity of MYOD and MYOG^22,23^. Given that TBX2 inhibits the ability of MYOG to activate transcription, we asked if TBX2 represses *TCEA3*. We used previously characterized shTBX2 constructs^23^ to deplete TBX2 in the RH30 cell line. Depletion of TBX2 was confirmed at the mRNA and protein level^24^. TBX2 depleted and scrambled control cells were assayed for mRNA expression of TCEA3 and we found that TCEA3 was upregulated upon TBX2 depletion (Fig. 2a). To ask if this effect was direct, we performed chromatin immunoprecipitation assays for TBX2 and found that TBX2 was enriched on the *TCEA3* promoter (Fig. 2b), indicating that TBX2 directly binds to the *TCEA3* promoter to repress expression. TBX2 has recently been shown to recruit heterochromatin protein 1 (HP1) and DNA methyltransferase 1 (DNMT1) to target genes^25^, so we asked if TCEA3 expression could be correlated with DNA methylation in cancer cell lines using the Cancer Cell Line Encyclopedia (CCLE) database. We found that TCEA3 expression was strongly correlated with DNA methylation (Fig. 2c). The data were highly consistent with the TCEA3 expression data shown here. *TCEA3* was highly repressed in the RH30 cell line, where the methylation signal was high. TCEA3 was more weakly expressed in the RD cell line, which correlated with lower levels of methylation. This correlation could be seen in a comparison with only soft tissue sarcoma cell lines (Supplementary Fig. 1A), but could also be seen in a comparison of all cancer cell lines (Fig. 2c), suggesting that DNA methylation silencing of *TCEA3* is a common mechanism in cancer cells. As a control, we also examined the methylation status of *TCEA1* and found that it was not correlated with methylation (Supplementary Fig. 1B). To determine if *TCEA3* was silenced by DNA methylation, the RH30 cell line was treated with the DNA methyltransferase inhibitor 5-aza-2′deoxycytidine. We found that *TCEA3* mRNA was upregulated and *TCEA1* mRNA was unaltered, consistent with the genomic methylation data (Fig. 2d). Taken together, the data show that *TCEA3* is silenced by DNA methylation and suggests a possible mechanism for how TBX2 represses *TCEA3* expression in RMS and additional cancer types.

### TCEA3 overexpression in RMS cell lines

**Fig. 3:**
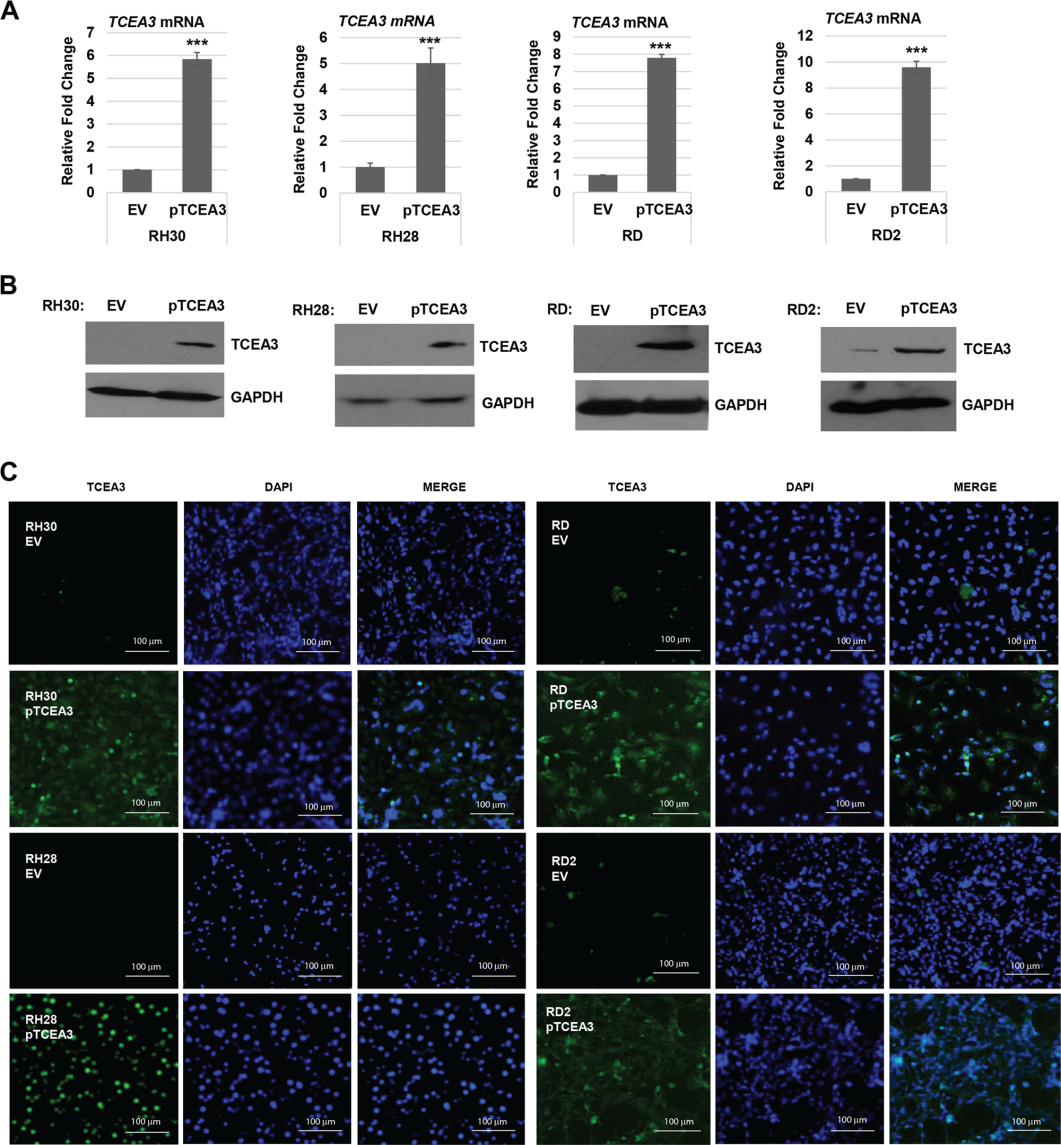
Exogenous TCEA3 overexpression in RMS cell lines. **a** RH30, RH28, RD, and RD2 cell lines were transfected with a TCEA3-expressiion plasmid (pTCEA3) or the empty vector (EV). TCEA3 ectopic expression was confirmed by assaying TCEA3 mRNA expression by qRT-PCR. **b** Western blot with antibodies against TCEA3 was performed on the cells described in (**a**). to confirm the protein expression of TCEA3. GAPDH was used as the loading control. **c** TCEA3 immunofluorescence with antibodies against TCEA3 and DAPI used to stain nuclei on cells described in (**a**). Scale bar is 100 µm. Error bars are S.E.M. Student's *t* test; ***p < 0.001 with respect to EV, *n* ≥ 3 biological replicates.

To understand how the restoration of TCEA3 would impact RMS cells, we ectopically expressed TCEA3 using a mammalian expression vector for TCEA3 (pTCEA3) in RMS cell lines representing both ERMS and ARMS subtypes. We confirmed the overexpression at the level of both RNA (Fig. 3a), and protein (Fig. 3b). TCEA3 has been described as a cytoplasmic factor^26,27^ and we have shown TCEA3 translocates from the cytoplasm to the nucleus upon differentiation^7^. To confirm the expression of TCEA3 and determine if it was present in the nucleus or cytoplasm, TCEA3 was detected by immunofluorescence in RMS cell lines stably expressing TCEA3 and we found that TCEA3 appeared to be expressed primarily in the nucleus (Fig. 3c). While TCEA3 was primarily in the nucleus, some cytoplasm localization could also be detected, particularly in the ERMS cell lines (Fig. 3c).

### TCEA3 overexpression inhibits proliferation of RMS cell lines

**Fig. 4:**
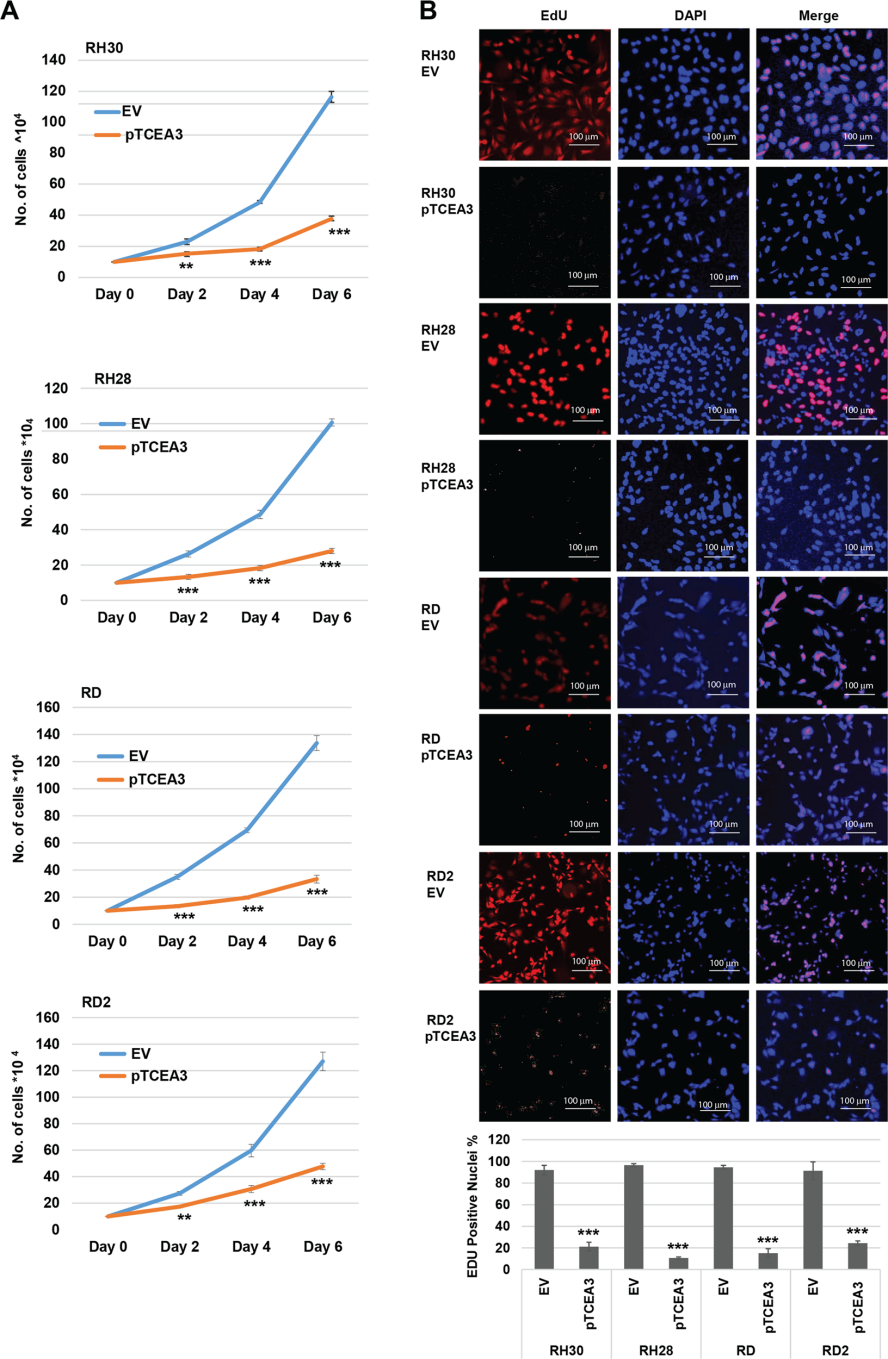
TCEA3 expression inhibits RMS cell line proliferation. **a** RMS cells stably expressing TCEA3 or the vector control (EV) were seeded at the same number of cells and harvested for cell counts every 2 days. **b** Proliferation of RMS cells expressing TCEA3 was assayed by an EdU cell proliferation assay. Blue (DAPI) represents nuclei, and red represents EdU labeled nuclei, which labels cells undergoing DNA synthesis (S phase) during the time window of EdU treatment. Scale bar is 100 µm. EdU positive nuclei were counted in at least five random fields in microscopic images and percentage was calculated in comparison to total nuclei in the same images. Error bars are S.E.M. Student's *t* test; **p < 0.01, ***p < 0.001 with respect to EV, *n* ≥ 3 biological replicates except proliferation assay (*n* = 2).

To determine if TCEA3 could inhibit proliferation of RMS cell lines, ARMS (RH30 and RH28) and ERMS (RD and RD2) cell lines expressing exogenous TCEA3 were assayed for proliferation and we found that these cells had reduced proliferation rates when compared with vector control (Fig. 4a). To determine if TCEA3 inhibited DNA synthesis, we quantitated newly synthesized DNA by measuring EdU incorporation and found that expression of TCEA3 inhibited the synthesis of new DNA in both ARMS and ERMS cell lines (Fig. 4b), suggesting that TCEA3 inhibits proliferation and DNA synthesis in RMS cell lines.

### TCEA3 impairs migration and mobility in RMS cell lines

To determine if TCEA3 regulates RMS cell migration, we performed a scratch assay and found that RH30 (ARMS) (Supplementary Fig. 2A) and RD (ERMS) (Supplementary Fig. 2B) cell lines with exogenous TCEA3 had reduced mobility when compared with the control cells. To assay the role of TCEA3 in tumorigenesis, we performed a soft agar assay to detect anchorage-independent growth. We found that ectopic overexpression of TCEA3 in RH30 (Supplementary Fig. 2C) and RD (Supplementary Fig. 2D) cell lines highly suppressed anchorage-independent growth compared to control cells, which readily formed colonies in soft agar. Thus, the results indicate that TCEA3 inhibits anchorage-independent growth, a hallmark of cell transformation.

### TCEA3 overexpression induces apoptosis

**Fig. 5:**
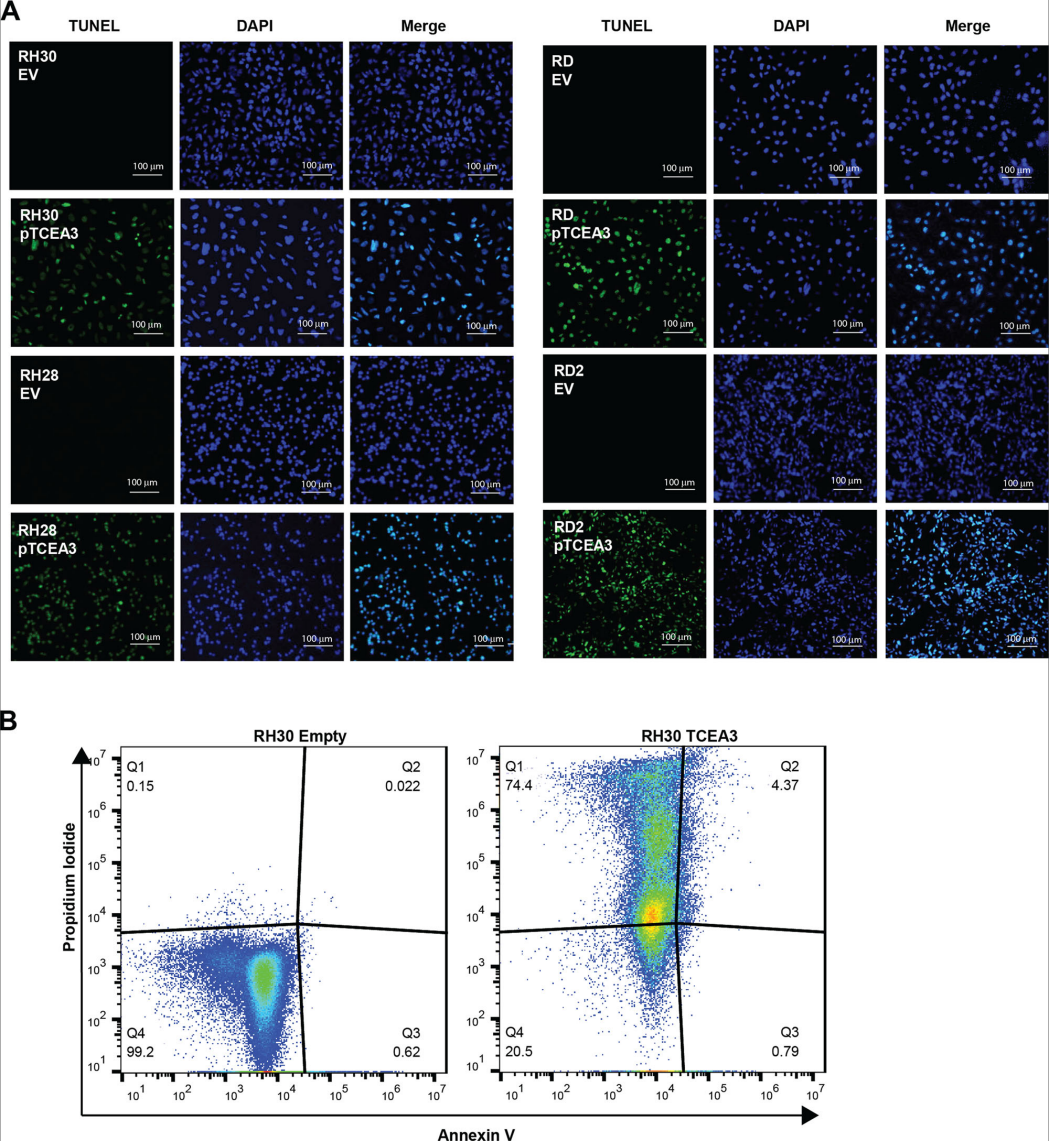
TCEA3 expression induces cell apoptosis. **a** RMS cell lines expressing TCEA3 have increased cell apoptosis as shown by TUNEL assay. Scale bar is 100 µm. **b** PI/Annexin V analysis of apoptosis in the RH30 cell line expressing TCEA3 or empty vector control. The flow cytometry profile represents Annexin V and Propidium iodide staining along the *X* and *Y* axis, respectively. The values shown in the lower left, lower right, upper right and upper left quadrants of each panel represent the percentage of live, early apoptotic, late apoptotic and dead cells, respectively.

Together, our results showed that exogenous expression of TCEA3 in RMS cell lines inhibited proliferation, anchorage-independent growth and impaired migration and mobility. Given the severe reduction in proliferating cells we observed, we asked if the TCEA3 expressing cells were undergoing apoptosis. A TUNEL assay was performed to check for apoptotic cells undergoing extensive DNA fragmentation during the late stages of apoptosis. TUNEL assays revealed a dramatic increase in both ERMS and ARMS cell lines transfected with TCEA3 (Fig. 5a) compared to the controls. To confirm that the TCEA3 expressing cells were undergoing apoptosis, apoptotic cells were detected by flow cytometry using Annexin V and propidium iodide in RH30 cell lines expressing TCEA3 or vector control. We found that exogenous expression of TCEA3 increased the percentage of apoptotic cells detected and also dramatically increased the percentage of dead cells present (Fig. 5b). The degree of dead cells present (74.4%) was consistent with the high frequency of TUNEL^+^ cells and it is important to note that these cells could not be maintained in culture beyond four to six passages due to the high frequency of cell death.

### Cross talk between the intrinsic and extrinsic pathway is involved in the apoptosis induced by TCEA3 overexpression

**Fig. 6:**
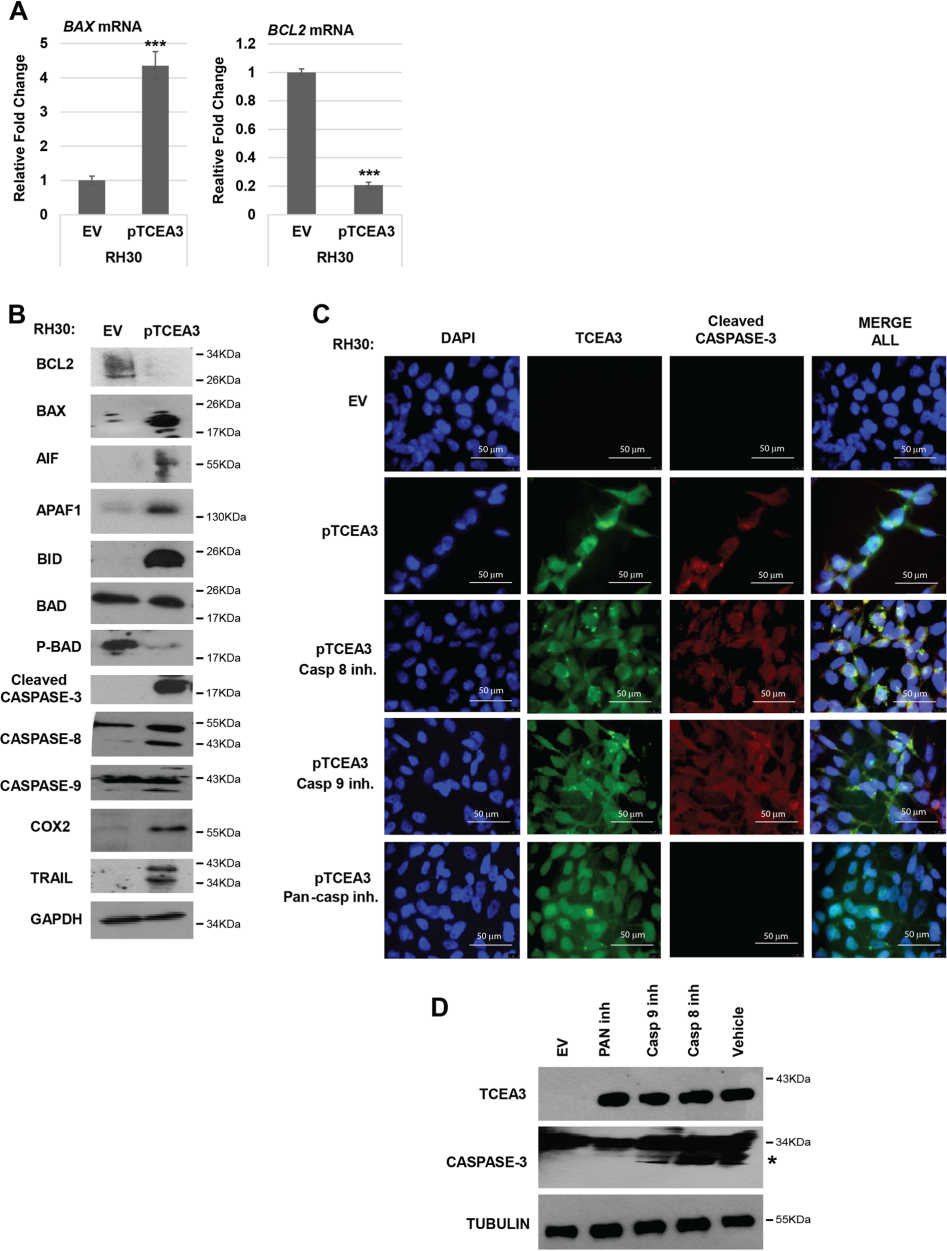
Characterization of apoptosis triggered by TCEA3 expression. **a** RH30 cells expressing TCEA3 or empty vector control were harvested for RNA. *BAX* and *BCL2* gene expression were assayed by qRT-PCR. Scale bars 50 µm. **b** RH30 cells expressing TCEA3 or empty vector were assayed by western blot with antibodies against the indicated apoptotic marker proteins. GAPDH was used as a loading control. **c** RH30 cells expressing TCEA3 and empty vector were treated with caspase 8 inhibitor (Z-IETD-FMK, 40 μM), caspase 9 inhibitor (Z-LEHD-FMK, 40 μM) or a pan-caspase inhibitor (Z-VAD-FMK, 50 μM) for 18 h. Immunofluorescence assay was done with anti TCEA3 antibody (green) and anti-cleaved caspase 3 antibody (red). DAPI (blue) was used to visualize nuclei. Scale bar is 50 µm. **d** Western blot assay on the same cells as in C. to confirm the protein expression of TCEA3, caspase 3 and tubulin as a loading control. * marks cleaved caspase 3. Error bars are S.E.M. Student *t* test; ***p < 0.001 with respect to EV, *n* ≥ 3 biological replicates.

To understand how TCEA3 promoted apoptosis, we assayed for gene expression changes associated with apoptosis in RH30 cell lines expressing TCEA3 or vector control. We initially examined mRNA expression of the anti-apoptotic gene, *BCL2*, and the pro-apoptotic regulator, *BAX*. We found that *BCL2* was downregulated and *BAX* was upregulated at the mRNA level (Fig. 6a). The downregulation of BCL2 and the upregulation of BAX was confirmed at the protein level as well (Fig. 6b). Given these results, we examined the expression changes of other known mediators of apoptosis. Apoptosis Initiating Factor (AIF), APAF-1 (Apoptotic protease activating factor 1), and BID (BH3 interacting domain death agonist) were examined and we found that each of these proteins were highly upregulated upon the expression of TCEA3. We also examined the modification of BAD (Bcl-2 associated death promoter), a pro-apoptotic member of the BCL-2 gene family involved in initiating apoptosis. BAD is regulated by phosphorylation and dephosphorylated BAD is an inducer of apoptosis. We found that total levels of BAD were relatively unchanged, but the phosphorylation of BAD was strongly inhibited upon TCEA3 overexpression (Fig. 6b). We next examined the caspase family including caspase-3, caspase-8 and caspase-9 and found that cleaved caspase products could be seen for each of these caspases in TCEA3 overexpressing cells. We also found the cyclooxygenase 2 (COX2) was upregulated. TNF-related apoptosis inducing ligand (TRAIL) was also upregulated in TCEA3 expressing cells. The cleavage of both caspase-8 and caspase-9 was surprising, as it indicated that both the intrinsic and extrinsic pathway were activated upon TCEA3 expression.

The extrinsic and intrinsic pathways can induce apoptosis independently but they can also cross talk and induce apoptosis^28^. Our results showed that both caspase-8 and 9 are cleaved upon overexpression of TCEA3, indicating that both the extrinsic and intrinsic pathways are activated. To determine which caspase pathway was required to activate apoptosis upon exogenous TCEA3 expression, we treated the RH30 cell line with pTCEA3 with intrinsic, extrinsic or pan-caspase inhibitors. Apoptosis was detected by examining the cleavage of caspase-3, which is activated by both the extrinsic and intrinsic pathways. We found that cleaved caspase-3 could be detected after inhibition of either the intrinsic or extrinsic caspases, but the pan-caspase inhibitor blocked caspase-3 cleavage (Fig. 6c). To confirm this result, caspase-3 was also detected by western blot assays following caspase inhibitor treatment. This result confirmed that only pan-caspase inhibitors could block caspase-3 cleavage (Fig. 6d). Together, the results show that TCEA3 promotes caspase dependent apoptosis through both the intrinsic and extrinsic pathways.

### TCEA3 is under expressed in many cancer types and can initiate apoptosis in other cancer cell lines

**Fig. 7:**
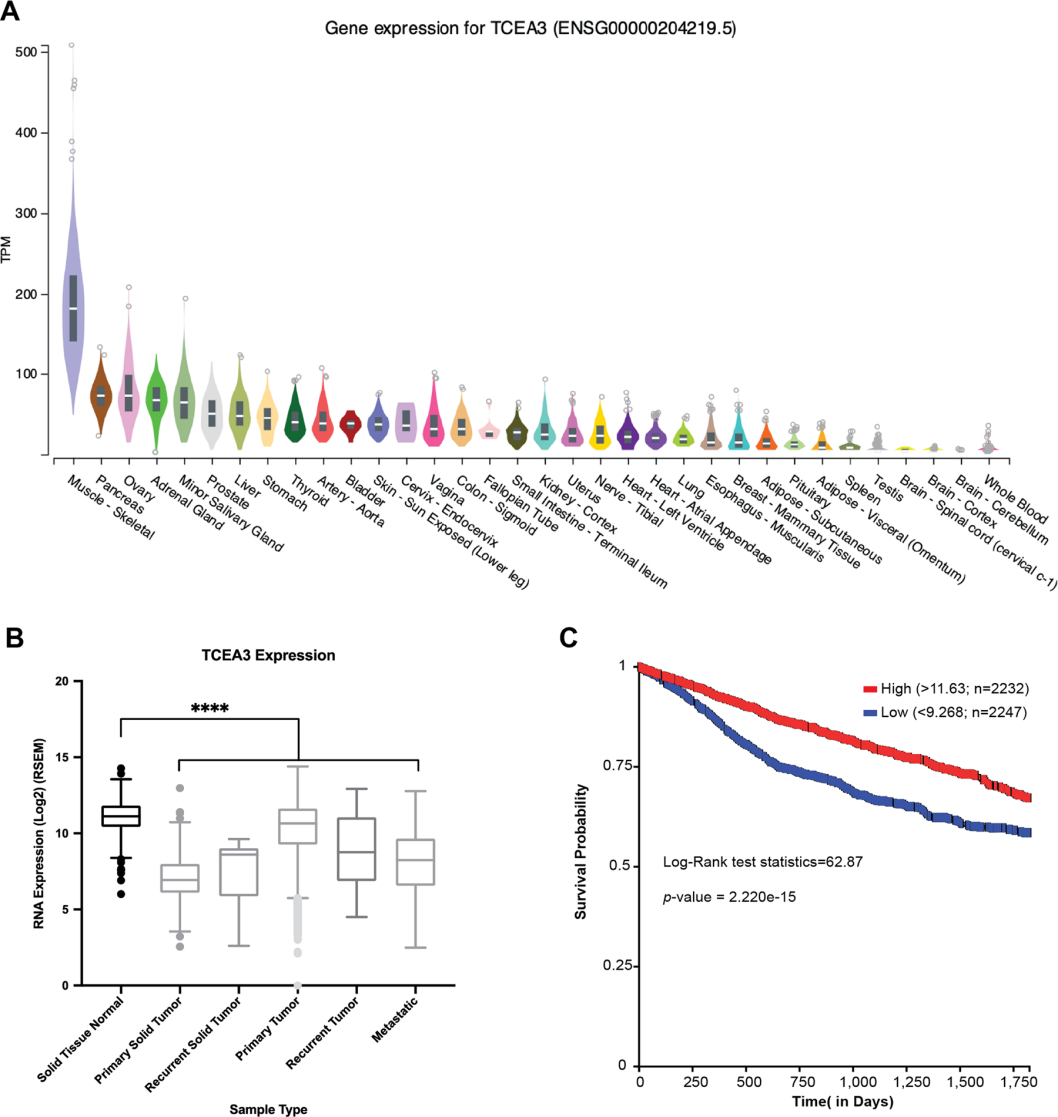
TCEA3 is highly expressed in normal skeletal muscle and has lower expression across all cancers. **a** TCEA3 is most expressed in skeletal muscle. RNA expression of TCEA3 was analyzed and downloaded from the genomic-tissue expression (GTEx) database for different tissue types. The transcripts per million (TPM) was plotted on a linear scale. **b** Low expression of TCEA3 is associated with cancer. TCEA3 expression across the various cancer sample types along with normal solid tissue from GTEx, TCGA, and TARGET databases were analyzed. (*n* = 10660, ANOVA followed by Bonferroni multiple comparison test, ****p < 0.0001). **c** Patients with lower expression of TCEA3 have poorer survival. Kaplan Meier (KM) 5-year survival analysis was plotted for “primary tumor” samples downloaded from the TCGA and TARGET cancer database using UCSC Xena tool. The samples were divided in two non-overlapping groups based on TCEA3 expression: High (Red, *n* = 2232) with RNA expression (log2 transformed) greater than 11.63 (top 25%), and Low (Blue, *n* = 2247) with RNA expression (log2 transformed) less than 9.268 (bottom 25%) (*N* = 9185, Log-Rank test statistic = 62.87, *p* = 2.22-e-15).

We have shown that TCEA3 is expressed in skeletal muscle where it promotes myogenesis^7^. However, we show here the surprising finding that TCEA3 promotes apoptotic cell death in RMS cell lines. TCEA3 is known to be tissue restricted^5^, but the expression of TCEA3 in various tissues has not been extensively characterized. To gain insight into the expression of TCEA3 in other organ systems, we examined gene expression profiles in the Genotype-Tissue Expression (GTEx) database. We saw that skeletal muscle showed the highest expression of TCEA3 (Fig. 7a), in agreement with our previous work^7^. However, expression of TCEA3 could also be detected in many other tissues, including prostrate, ovary, cervix and breast. We also asked if TCEA3 was downregulated in broad cancer types and we found that TCEA3 was downregulated in all tumor types analyzed (Fig. 7b). Finally, the expression of TCEA3 in cancer was correlated with lifespan and we saw that patients with higher expression of TCEA3 had a significantly higher five-year survival rate (Fig. 7c). These data suggested that TCEA3 might commonly be suppressed in cancer to block apoptosis. To test this hypothesis, we examined the expression of TCEA3 in HeLa, PC3, MCF7, and MDA-321 cell lines representing cervical, prostate and breast cancer, respectively. We found that TCEA3 could not be detected in any of these cell lines (Supplementary Fig. 3). We ectopically expressed TCEA3 in each of these cell lines and expression was confirmed by immunofluorescence (Supplementary Fig. 3). As we observed in RMS cell lines, we detected primarily nuclear staining of exogenous TCEA3 (Supplementary Fig. 3). We next assayed for DNA synthesis by EdU incorporation and found that TCEA3 inhibited EdU incorporation in each cell line tested (Supplementary Fig. 4). Finally, we assayed for apoptosis by TUNEL assays and found that each of these cell lines initiated apoptosis in response to TCEA3 (Supplementary Fig. 5). These results suggest that there is a common role of TCEA3 in each of these cell types that allows TCEA3 to trigger apoptosis and that this may be a common phenomenon in many cancer types.

### TCEA3 sensitizes RMS cell lines to chemotherapeutic drugs

**Fig. 8:**
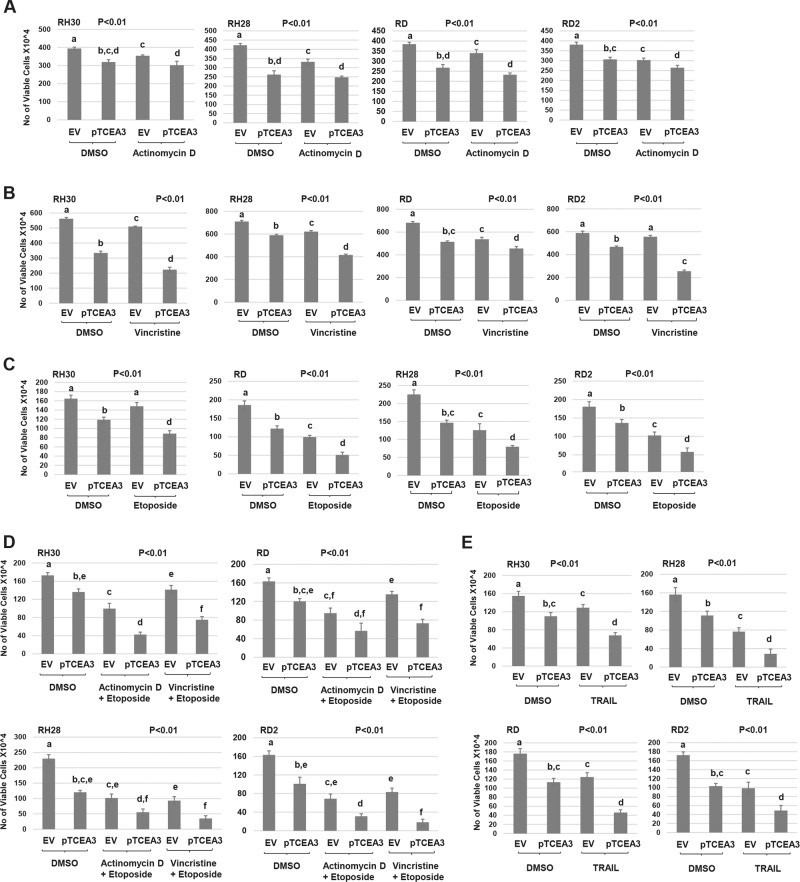
Overexpression of TCEA3 modulates RMS cell line sensitization to anticancer drugs. **a** RH30, RH28, RD, and RD2 cell lines expressing TCEA3 (pTCEA3) or empty vector (EV) were seeded at 30 × 10^4^ per well in 6-well plates. After recovery for 24 h, cells were treated with 10 nM actinomycin D for 48 h. Viable cells were counted by trypan blue assay. **b**. TCEA3 overexpression sensitizes RMS cell lines to vincristine. Cells as in (**a**). were treated with 10 nM vincristine for 3 days, and then viable cells were counted by trypan blue assay. **c** TCEA3 overexpression sensitizes RMS cell linea to etoposide. Cells as in A. were treated with 50 μM etoposide for 24 h and viable cells were counted by trypan blue assay. **d** TCEA3 expression sensitizes RMS cell lines to anticancer drug combinations. Cells as in (**a**) were treated with combination of 10 nM actinomycin D with 50 μM etoposide or 10 nM vincristine with 50 μM etoposide for 24 h, and viable cells were counted using trypan blue. DMSO was used as a negative control. **e** RMS cell lines as in (**a**) were treated with TRAIL at 0.5 µg/ml. After 12 h, cells were trypsinized for trypan blue assay. Data are plotted as means with S.E.M. and analyzed with one-way ANOVA followed by Tukey’s HSD post-hoc test. Means not sharing same letter are statistically significant. *n* ≥ 3 biological replicates.

We next asked if exogenous expression of TCEA3 in RMS cell lines could sensitize these cells to commonly used chemotherapeutic drugs. We first used actinomycin-D (Dactinomycin), which is an antitumor antibiotic that blocks nucleic acid synthesis. RMS cell lines representing both ERMS and ARMS expressing exogenous TCEA3 and vector control were treated with actinomycin-D and cell viability was assayed (Fig. 8a). We found that exogenous TCEA3 only mildly sensitized RD2 (ERMS) cells to actinomycin-D and the other cell lines showed no effect. The second drug tested was vincristine (vincristine sulfate) which serves as the backbone for the majority of chemotherapeutic regimes for RMS^29^. Vincristine can bind to microtubular proteins of the mitotic spindle and inhibit polymerization which leads to metaphase arrest. RMS cell lines expressing exogenous TCEA3 and vector control were treated with vincristine and cell viability was assayed (Fig. 8b). We found that expression of TCEA3 sensitized each of these cell lines representing both ERMS and ARMS to vincristine. The third treatment used was etoposide (etoposide phosphate), which has the ability to inhibit DNA topoisomerase II, thus inhibiting DNA synthesis and causing DNA damage. RMS cells expressing exogenous TCEA3 and vector control were treated with etoposide and cell viability was assayed (Fig. 8c). We found that, like the results with vincristine, TCEA3 sensitized cells to etoposide in each cell line tested. To extend these results, we combined two chemotherapeutic drugs together to test for synergy. It should be noted that due to the high level of cell death present in both control and TCEA3 expressing cells, the incubation time was much shorter in these experiments. We found that TCEA3 expression sensitized RH30 and RH28 cell lines (ARMS) to both combinations tested (Fig. 8d). A statistically significant sensitization was also found in the RD2 cell line but not in the RD cell line (Fig. 8d). Finally, we treated the cells with TRAIL (TNF-Related Apoptosis Inducing Ligand), a peptide which promotes apoptosis in cancer cells, although not all cancer cells are susceptible to TRAIL^30,31^. RMS cells expressing exogenous TCEA3 and vector control were treated with TRAIL and we found that TCEA3 expression sensitized each of the cell lines to TRAIL (Fig. 8e).

## Discussion

In this work, we show that TCEA3 is severely downregulated in cell lines representing both subtypes of RMS. Exogenous expression of TCEA3 promotes apoptosis in a caspase-dependent manner though both the intrinsic and extrinsic pathways. Our results were highly unexpected, as TCEA3 is robustly expressed in skeletal muscle upon differentiation, where it promotes the activity of MYOD and MYOG by promoting RNAPII recruitment and elongation on MRF dependent genes. Thus, we anticipated that TCEA3 would promote differentiation in RMS cells. However, the strong cell proliferation defect noted in both ERMS and ARMS cell lines suggested that TCEA3 might have another function in RMS. Our results showing that TCEA3 promoted apoptosis in both ERMS and ARMS cell lines were surprising, but supported by studies in both ovarian and gastric cancer. In ovarian cancer cell lines, TCEA3 has been shown to induce Smad-independent, JNK-dependent apoptosis^26^. In gastric cancer cell lines, TCEA3 is downregulated with respect to normal tissue and exogenous expression promotes apoptosis^32^. Thus, our results are consistent with other studies and suggest that there is a shared function of TCEA3 in several cancer types. A mechanistic basis for how TCEA3 promotes apoptosis and functions as a tumor suppressor is not yet understood. The dysregulation of transcriptional programs is a well-known feature of cancer biology^33^. However, other elongation factors, such as the Super Elongation Complex (SEC), have been shown to promote tumor growth and inhibitors of these complexes had been shown to inhibit tumor growth^34^. The oncogene MYC is a target of SEC and SEC inhibition downregulates MYC and the MYC dependent transcriptional program^34^. Both SEC and the TFIIS family are transcription elongation factors, but their biochemical activities are distinct. Both function to promote productive elongation by RNAPII, but SEC functions to release promoter-proximal paused RNAPII and TFIIS functions to release arrested RNAPII by promoting cleavage of the nascent transcript to resume transcription^35^. TCEA3 is particularly interesting as it is tissue restricted and expressed in both the cytoplasm and nucleus. We have shown that upon skeletal muscle differentiation, TCEA3 translocates to the nucleus, where it enhances the recruitment of RNAPII to promoters and travels with elongating RNAPII. In the cytoplasm, TCEA3 interacts with Annexin A1^27^. Annexin A1 is recruited to cell membranes by dysferlin and promotes sarcolemma repair^36^. Annexin A1 also enhances TGF-β-Smad signaling to induce cell migration and enhances metastasis formation of basal-like breast cancer cells^37^. The function of Annexin A1 in RMS is uncharacterized. It will be important to understand the contribution of the cytoplasmic and nuclear functions of TCEA3 that mediate the induction of apoptosis. We show that TCEA3 sensitizes RMS cell lines to chemotherapeutic drugs, suggesting that reactivating *TCEA3* in cancer cells is an attractive novel therapeutic target for RMS treatment that would specifically initiate apoptosis in cancer cells while sparing normal cells. We also show that *TCEA3* is regulated by TBX2 and DNA methylation and it will be important to determine if TBX2 directs DNA methylation of the *TCEA3* promoter. TCEA3 has also been shown to be downregulated upon deletion of MLL3, a subunit of the COMPASS complex required for H3K4 monomethylation at gene enhancers^38^. The polycomb (PRC) complex and the COMPASS complex control the repressed and activated steps of gene expression, respectively. It has been proposed that the balance between these complexes is required for normal cellular function, and disruptions in this balance leads to pathology^39^. In RMS, the catalytic subunit of PRC2, EZH2, is known to be upregulated and required for protecting ARMS from apoptosis^40^ and driving cancer growth in ERMS^41,42^. We have recently shown that EZH2 also serves to maintain the expression of TBX2 in RMS, by repressing the TBX2 repressor, TBX3^24^. It will be important to further characterize the function and regulation of TCEA3 to therapeutically harness this potent tumor suppressor and improve treatment strategies for RMS and other cancers.

## Materials and methods

### Cell culture

RD, RH30, HeLa, MCF7, MDA-MB 321, and PC3 cells were obtained from ATCC. RD2, and RH28 were obtained from Dr. Denis Guttridge (Medical University of South Carolina). RD, RD2, RH30, RH28, HeLa, MCF7 and MDA-MB 321 were grown according to standard protocols in Dulbecco’s modified Eagle medium (DMEM) supplemented with 10% Fetal Bovine serum (FBS)(Hyclone) and penicillin and streptomycin antibiotics. PC3 cells were grown in RPMI basal media with L-Glutamine supplemented with 10% FBS and penicillin and streptomycin antibiotics. Proliferating C2C12 myoblasts (ATCC) were grown in DMEM supplemented with 10% fetal bovine serum (Hyclone). All cells were grown at 37 ^o^C in a CO_2_ incubator at 5% CO_2_. Bio-Synthesis authenticated all RMS cell lines (Lewisville, TX) using STR analysis on September 14, 2011.

### Cloning

Murine TCEA3 was PCR amplified from cDNA reverse transcribed from RNA isolated from C2C12 cells differentiated for four days. PCR amplified fragments were cloned into the pEF6/V5 His TOPO TA expression vector (Invitrogen) according to the manufacturer’s protocol and clones were confirmed by sequencing.

### Cell transfections

Cells were transfected with the TurboFect Transfection Reagent (Thermo Scientific) according to manufacturer’s protocol. To generate stable cell lines, cells harboring pTCEA3 or pEF6 (EV) were selected for using blasticidin (10 µg/ml). Once confirmed, cell lines were frozen and stored in liquid nitrogen vapor. Aliquots were thawed and used for each experimental approach shown as cells could not be indefinitely maintained in culture due to the frequency of cell death. Cells were passaged no more than four times for all experimental data shown.

### Quantitative real time PCR

RNA was extracted from cells using Trizol (Life Technologies, Carlsbad, CA) and treated with DNase (Promega, Madison, WI). Two microgram of total RNA was reverse transcribed with MultiScribe TM MuLV reverse transcriptase (Life Technologies, Carlsbad, CA) and 40 ng of cDNA was used for quantitative real-time polymerase chain reaction (qRT-PCR) amplification (Life Technologies, Carlsbad, CA) with SYBR green PCR master mix (Life Technologies, Carlsbad, CA). Negative controls were included in samples where no reverse transcriptase was added for each RNA sample. The relative gene expression levels were normalized according to 18 S (F 5′ CGCCGCTAGAGGTGAAATTCT and R 5′ CGAACCTCCGACTTTCGTTCT) and/or HPRT1 (F 5′ TGACACTGGCAAAACAATGCA 3′ and R 5′ GGTCCTTTTCACCAGCAAGCT 3′). Primers used included TCEA3 F 5′TGTCCTTGGCCAAAGTCC and R 5′GGAGAAAGGCCTGCTTCTG, TCEA1 (F 5′ GAATGACAGCAGAGGAAATGG 3′ and R 5′ CATTGGTTCTTCAGCACTACG 3′), BAX F 5′ GCTGCAGAGGATGATTGC and R 5′ CCTTGAGCACCAGTTTGC and BCL2 F 5′ TGGCCTTCTTTGAGTTCG and R 5′ TCCGTTATCCTGGATCCA. Quantitative real-time reverse transcriptase PCR (qRT-PCR) data were calculated using the comparative Ct method (Life Technologies, Carlsbad, CA). Standard deviations were calculated from the mean of the ΔCt values calculated from at least two independent RNA samples.

### Western blot

Phosphate-buffered saline (PBS) washed cells were lysed in RIPA buffer supplemented with protease inhibitors (Complete, Roche Diagnostics, Indianapolis, IN) and centrifugation was used to obtain clear lysates. Bradford’s assay (Bio-Rad, Hercules, CA) was used to determine protein concentration. Fifty microgram protein was loaded in each well of sodium dodecyl sulfate polyacrylamide gel electrophoresis (SDS-PAGE). Resolved proteins were transferred onto a PVDF membrane using a tank blotter (Bio-Rad, Hercules, CA). Membranes were blocked with 5% milk in 1X Tris-buffered saline plus Tween 20 (TBST) and followed by incubation with primary antibody for overnight at 4 ^o^C. 1X TBST was used for washing membranes prior to incubation with the corresponding secondary antibody. Membranes were again washed with 1X TBST and incubated with chemiluminescent substrate according to the manufacture’s protocol (SuperSignal, Pierce, Rockford, IL) and visualized by autoradiography. TCEA3 protein was detected using anti-TCEA3 (R18, SCBT) antibody that recognizes both mouse and human proteins, which are predicted to have similar molecular weights. The other antibodies used included anti-GAPDH (Millipore), anti-BCL2 (D17C4, Cell Signaling), anti-BAX (2772S, Cell Signaling), anti-BAD (9292S, Cell Signaling), anti-p-BAD (Ser136)(4366P, Cell Signaling), anti-AIF (SCBT), anti-APAF1 (SCBT), anti-BID (SCBT), anti-COX 2 (SCBT), anti-caspase 9 (9502, Cell Signaling), anti-cleaved caspase-3 (Asp175) (5A1E, Cell Signaling), anti-caspase 3 (D3RGY, Cell Signaling), anti-caspase 8 (1C12) (9746 S, Cell Signaling), anti-TRAIL (SCBT) and anti-Tubulin (Developmental Studies Hybridoma Bank).

### Chromatin immunoprecipitation (ChIP)

ChIP assays were performed as described previously^43^. Anti-TBX2 (Abclonal) antibody was used for the immunoprecipitation (IP). Rabbit IgG (SCBT) was used as a non-specific control. Primers (F: 5′-CTGCGCTCTGGCCAGGAC-3′ and R: 5′- GTGCGTCAGGAGCGGTTC-3′) spanning *TCEA3* promoter were used for the real-time PCR. The real-time PCR was performed in triplicate. The results were represented as the percentage of IP over input signal (% Input). ChIP assays shown are representative of three individual experiments. Standard deviation from the mean was calculated and plotted as the error bar.

Enrichments were compared at a gene desert chromosomal region using primers (Chr 19F 5′ TCCGTTATCCTGGATCCA and 5′ CTTTGGTTGCCTGTGCTT).

### EdU staining assay

The Click-iT EdU Alexa Fluor 488 imaging kit (Life Technologies, Carlsbad, CA) was used to quantitate DNA synthesis. The kit was used according to manufacturer’s protocol. Nuclear staining was performed with DAPI (4′,6-diamidino-2-phenylindole, 1 μM, Life

Technologies, Carlsbad, CA) for 5 min at room temperature. At least five random fields were used to count the EdU positive nuclei on microscopic images taken at ×200 magnification using a Leica Microscope.

### Immunofluorescence Staining

Cells were grown on coverslips and fixed with 4% formaldehyde. Cells were permeabilized and blocked by incubating with goat serum and 1.0% NP-40 for 1 h. Primary antibodies were added and incubated at room temperature for 2 h. Coverslips were washed three times with 1X PBS, then secondary antibodies conjugated with Alexa Flour-488 goat anti-mouse or anti-rabbit antibodies (1:500, Life Technologies, Carlsbad, CA) were incubated at room temperature for 2 h in the dark. DAPI (1 μM, Life Technologies, Carlsbad, CA) was used to stain cell nuclei.

### Drug treatment

Cells were seeded in triplicate in a 6-well plate with 30 × 10^4^ cells per well and allowed to recover for 24 h before drug treatment. Cells were treated with caspase-8 inhibitor (Z-IETD-FMK, FMK007, R&D Systems), caspase-9 inhibitor (Z-LEHD-FMK, FMK008, R&D Systems) or a pan-caspase inhibitor (Z-VAD-FMK, ALX-260-020-M001, Enzo Life Science) at 40 μM, 40 μM or 50 μM, respectively, for 18 h. Cells were treated with either 10 nM vincristine (Cayman Chemical) and incubated for 72 h, 10 nM actinomycin D (Cayman Chemical) for 48 h, 50 μM etoposide (Acros Organics) for 24 h or 0.5 µg/ml TRAIL (PHC1634,Gibco) for 12 h. For multidrug treatment, combinations of etoposide (50 μM) and vincristine (10 nM), or etoposide (50 μM) and actinomycin D (10 nM) were added to the cells and incubated for 24 h. Cell viability was determined using trypan blue staining, and cell number was counted in three different wells on blinded samples. For DNA methyltransferase inhibition assay, RH30 cells were treated with either DMSO (vehicle) or 5-aza-2′deoxycytidine (Sigma-Aldrich) at 30 µM and 60 µM for 48 h prior to RNA isolation. Culture medium supplemented with fresh drug was changed every 24 h. All assays were performed at least twice to confirm results.

### Soft agar assay

Soft agar assays were performed in 60 mm dishes, in which 2 ml of 0.7% Nobleagar (Affymetrix, Santa Clara, CA) in growth medium was overlaid with 2 ml of 0.35% agar in growth medium containing cells. Cells were counted using hemocytometer and from each clone (3 × 10^5^) cells were plated in triplicate. 1 ml of culture medium was added every five days to each plate and cells were grown at 37 °C for the indicated weeks. Colonies were counted in five random fields using a dissecting microscope,

### Proliferation assay

Cells were seeded in 6-well plates of 4 × 10^4^ cells per well and harvested for counting by hemocytometer on the indicated days. Cell viability was determined using trypan blue staining. Cell counting was performed in duplicate on blinded samples and experiments repeated at least twice.

### Scratch assay

Cell mobility was assayed by growing cells to 100% confluence and scraping the cell monolayer with a 10 μl pipet tip in a straight line. Cell debris was removed by washing. Marks were created near the scratch line to obtain the same field during the image acquisition. The plate was then placed in a CO_2_ incubator at 37 °C for indicated hours to take images of the scratch.

### Apoptosis assays

DNA fragmentation was detected with a Click-iT TUNEL Alexa Fluor^TM^ 488 imaging assay kit that was used according to manufacturer’s protocol. The dead cell apoptosis kit with Annexin V alexa fluor™ 488 & propidium iodide (PI) (Invitrogen) was used according to manufacturer’s protocol. The labeled cells were detected by a BD Accuri 6 flow cytometer. At least 20,000 events were recorded per sample and condition and analyzed by FlowJo software.

### Genomic expression and survival analysis

TCEA3 and TCEA1 gene expression (RNAseq) and DNA methylation status, reduced-representation bisulfite sequencing (RRBS), of the locus was analyzed across all the cancer cell lines available at the CCLE database using the webtool Plotly at portal: https://portals.broadinstitute.org/ccle.

Tissue wide TCEA3 RNA expression (Fig. 7a) were analyzed, sorted by median expression and downloaded from the Genotype-Tissue Expression (GTEx) database (https://gtexportal.org) on 08/26/2019. The RNA expression was represented as transcripts per million (TPM) and plotted on a linear scale. Using UCSC Xena Functional Genomics explorer (http://xena.ucsc.edu/)^44^, the gene expression of TCEA3 from GTEx, The Cancer Genome Atlas (TCGA) and The Therapeutically Applicable Research to Generate Effective Treatments (TARGET) cancer study databases were sorted based on sample type, filtered, and only the sample types represented in Fig. 7b were downloaded. The RNA expression (RSEM) were Log(2) transformed. The downloaded RNA expression data was imported in GraphPad Prism 8.0, analyzed and plotted as box-plot (Fig. 7b). Kaplan Meier Survival plot (5-year) was plotted for non-overlapping first (Low group, mRNA expression < 9.268, *n* = 2247) and third (High group, mRNA expression > 11.63, *n* = 2232) quartile samples based on TCEA3 expression for “primary tumor” sample types only from both the TCGA and TARGET cancer database using UCSC Xena tool.

### Statistics

Data are presented as means ± standard errors (S.E.). Statistical comparisons were performed using unpaired two-tailed Student’s *t* tests or one-way ANOVA followed by Tukey’s HSD (honest significant difference) post-hoc test. For one-way ANOVA analysis, all means not sharing the same letter were statistically significant. Probability value of <0.05 was taken to indicate significance. All experiments were performed at least three times (*n* ≥ 3 biological replicates) unless otherwise noted.

## Supplementary information

Supplemental Figures 1-5
