# Supplementary material for: The transcription elongation factor TCEA3 induces apoptosis in rhabdomyosarcoma
Source: Cell Death Dis. 2020 Jan 27;11(1):67. doi: 10.1038/s41419-020-2258-x (PMC6985194; doi:10.1038/s41419-020-2258-x)

**Supplemental Data Figure Legends**

**Supplemental Figure 1. TCEA3 repression correlates with DNA methylation.** A. Scatterplot for TCEA3 mRNA expression (RNAseq) and *TCEA3* locus methylation level (RRBS) was generated for soft tissue sarcoma cell lines using the Cancer Cell Line Encyclopedia (CCLE) database, and the plot was edited using Plotly tool. Each dot represents a cell line. mRNA expression value below 0 have no or negligible expression, and the DNA methylation levels values ranging from 0 to 1 denoting unmethylated to fully methylated locus respectively. B. Scatterplot for TCEA1 mRNA expression (RNAseq) and *TCEA1* locus methylation level (RRBS) was generated for all cancer cell lines using the Cancer Cell Line Encyclopedia (CCLE) database, and the plot was edited using Plotly tool. Each dot represents a cell line. mRNA expression value below 0 have no or negligible expression, and the DNA methylation levels values ranging from 0 to 1 denoting unmethylated to fully methylated locus respectively.

**Supplemental Figure 2. TCEA3 inhibits migration and anchorage-independent growth.** A-B. Monolayer of confluent RH30 (A) and RD (B) cells with TCEA3 or vector control were lightly scratched with a pipette tip. Contrast images were taken immediately after wounding (0 h), 12h, and 24 h following. C.-D. RH30 (C) and RD (D) cells expressing TCEA3 or vector control (EV) were used for soft agar colony formation assays. Image were taken at 100 X magnification and scale bars represent 20 μm. Colony formation were quantified by counting in five random fields (lower panels). Error bars are S.E.M. Student t-test; ***$p < 0.001$ with respect to EV, n = 3 biological replicates.

**Supplemental Figure 3. Exogenous TCEA3 expression in additional cancer cell lines.** HeLa, PC3, MCF7 and MDA231 cell lines were stably transfected with pTCEA3 or empty vector and used for immunofluorescence with antibodies against TCEA3 and DAPI was used to stain nuclei. Scale bar is 100 µm.

**Supplemental Figure 4. TCEA3 inhibits DNA synthesis in additional cancer cell lines.** HeLa, PC3, MCF7 and MDA231 cell lines were stably transfected with pTCEA3 or empty vector and assayed by an EdU cell proliferation assay. Blue (DAPI) represents nuclei, and red represents EdU labeled nuclei, which labels cells undergoing DNA synthesis (S phase) during the time window of EdU treatment. Scale bar is 100 µm.

**Supplemental Figure 5. TCEA3 induces apoptosis in additional cancer cell lines.** HeLa, PC3, MCF7 and MDA231 cell lines were stably transfected with pTCEA3 or empty vector were assayed by TUNEL assay. DAPI was used to stain nuclei. Scale bar is 100 µm.

## Supplemental Figure 1

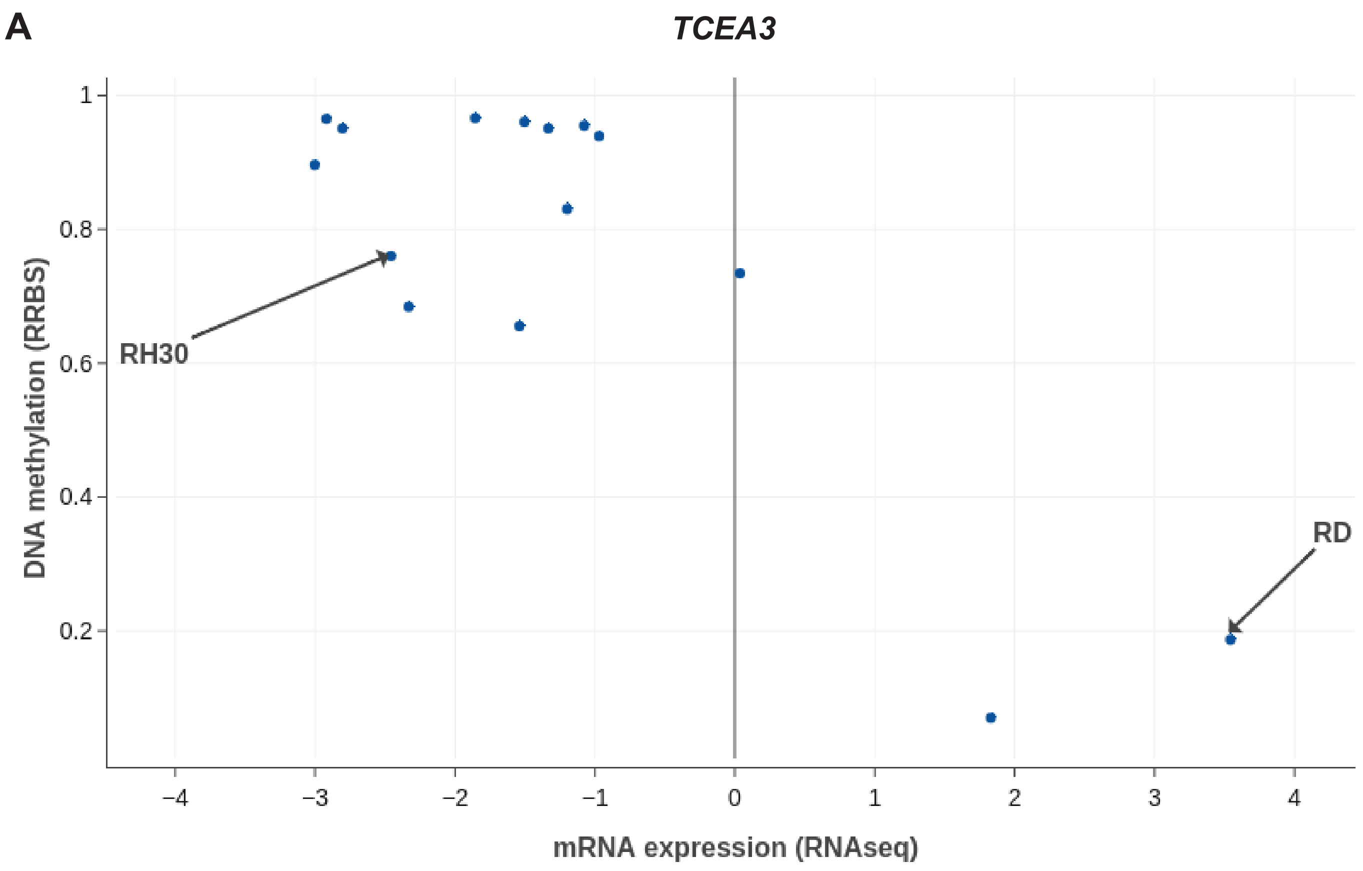


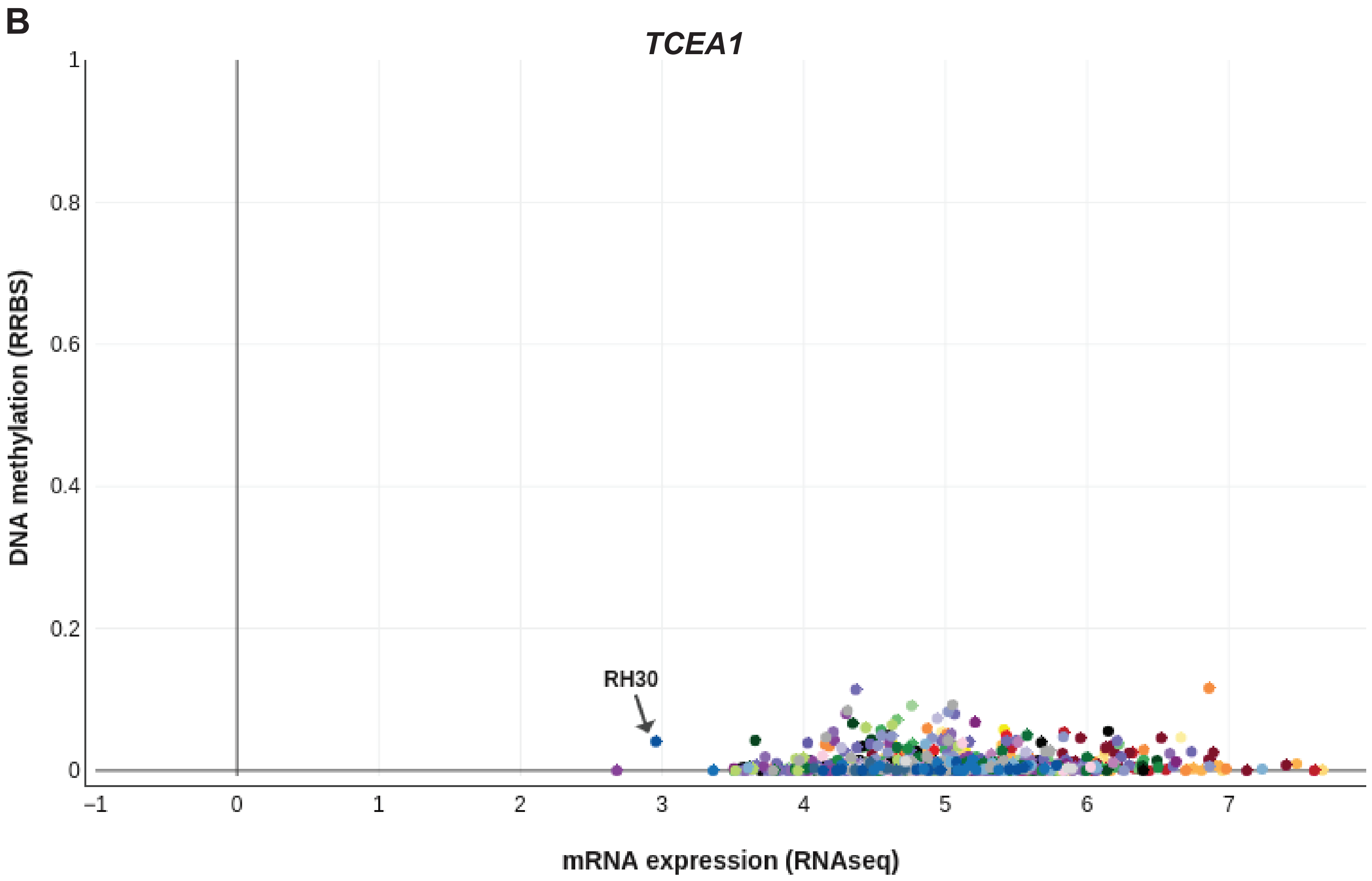

# Supplemental Figure 2

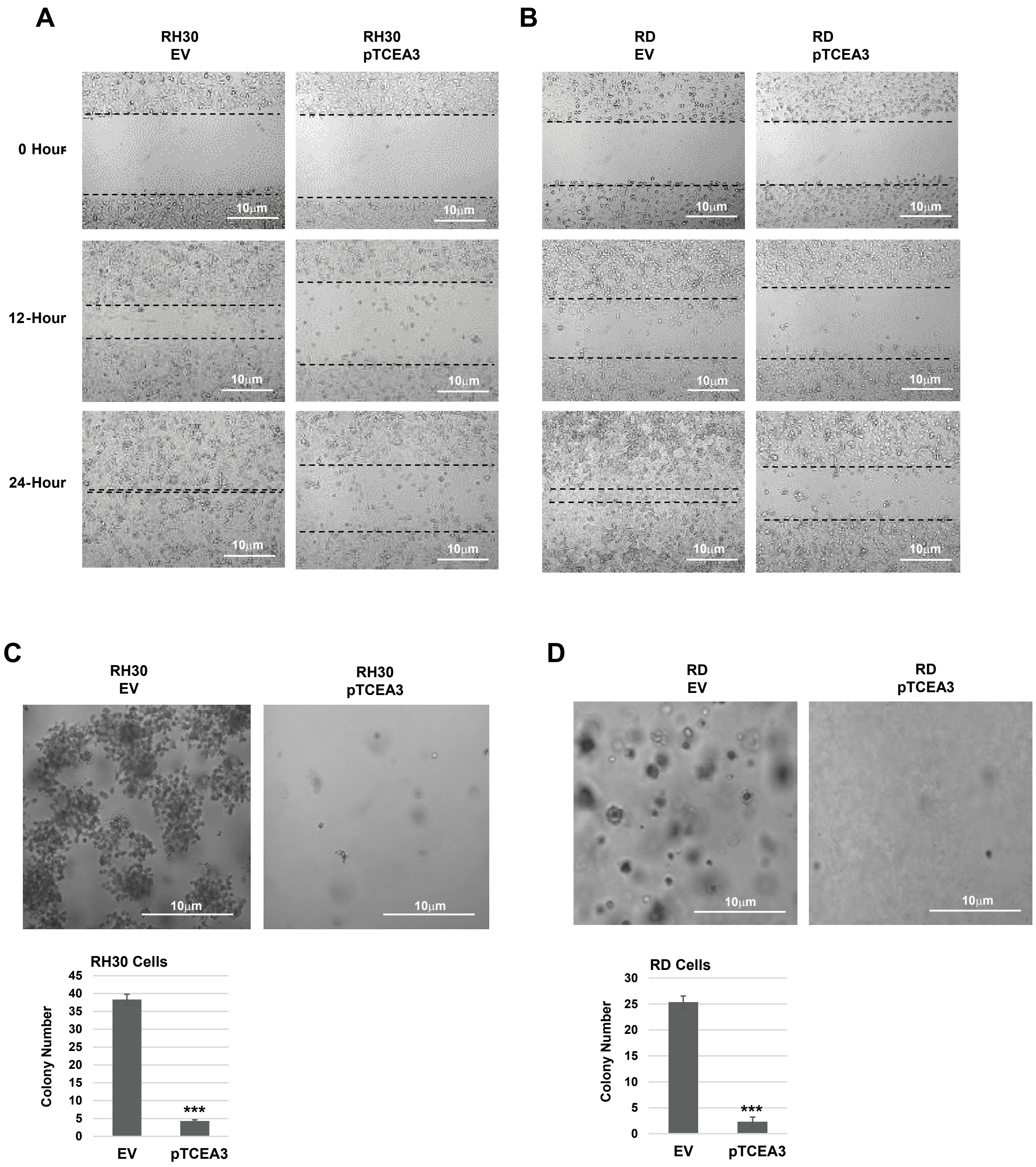

# Supplemental Figure 3

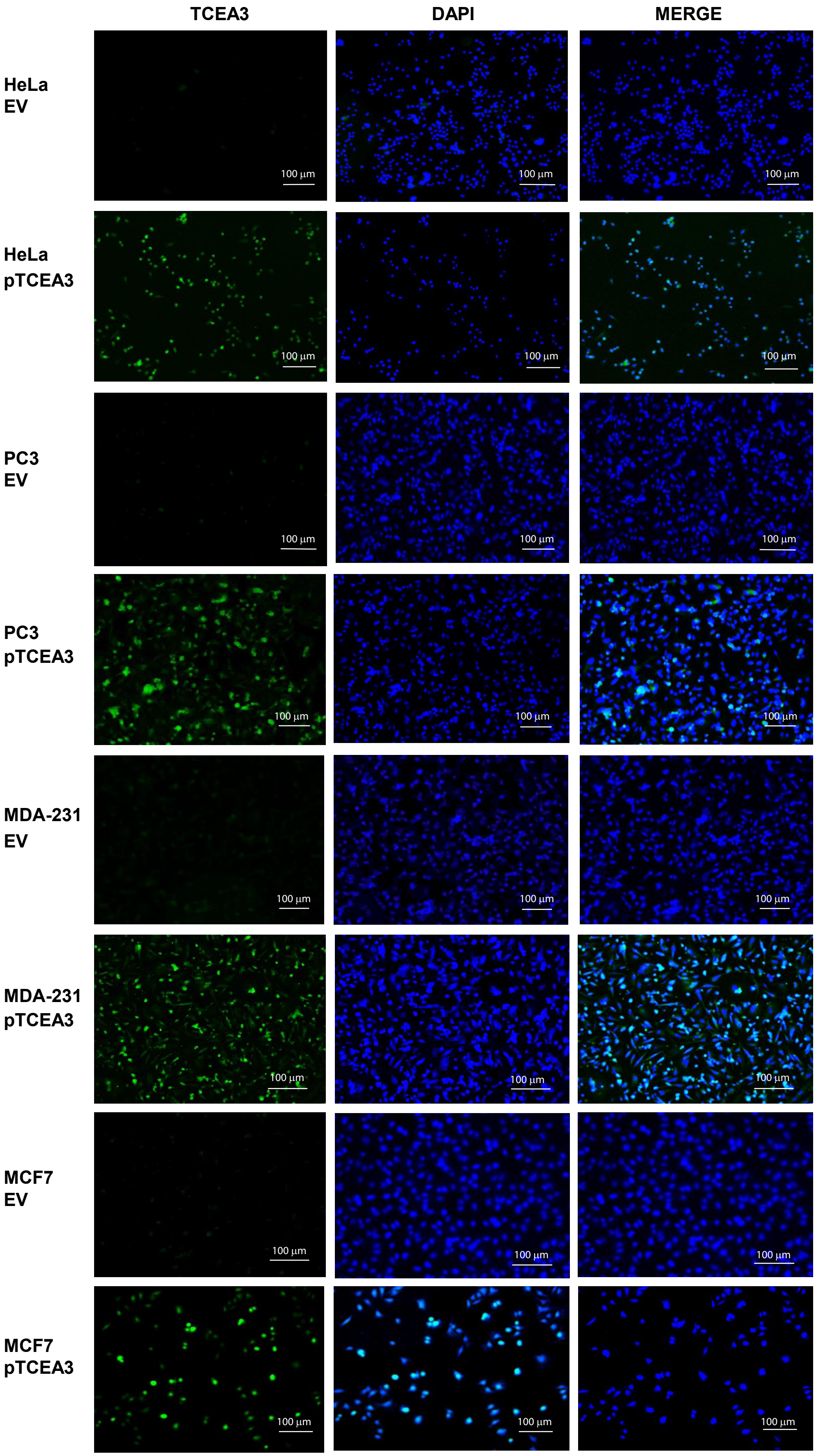

# Supplemental Figure 4

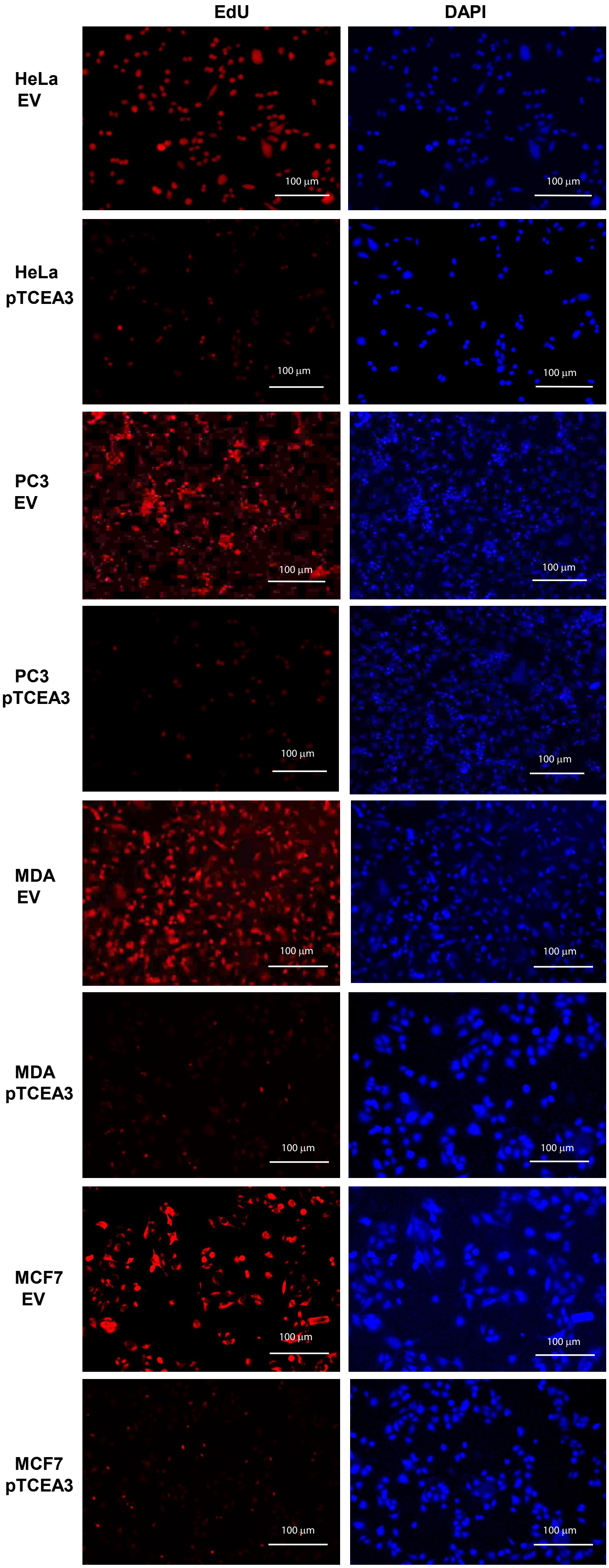

## Supplemental Figure 5

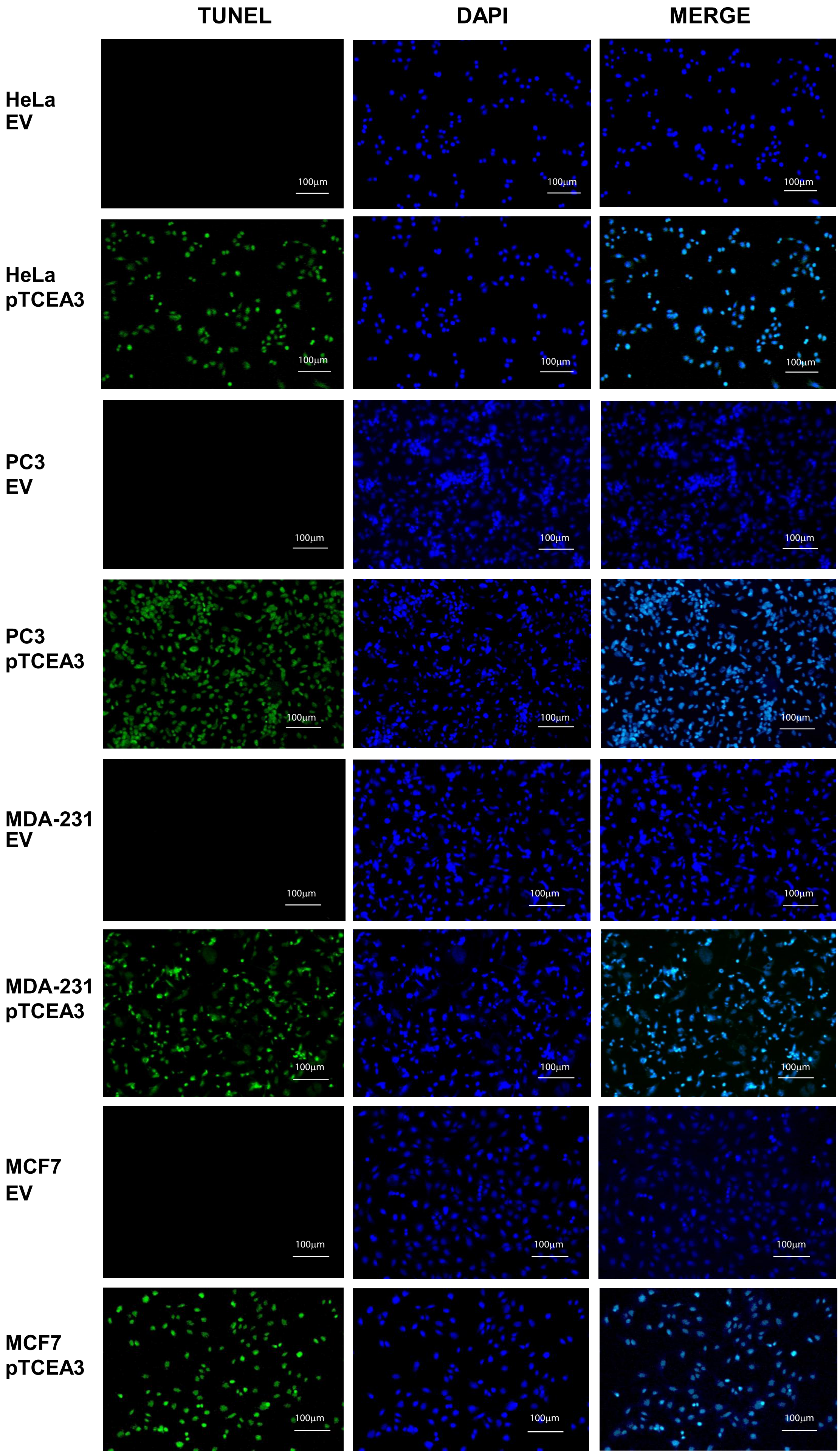

Supplement: Supplementary file 1 — Supplemental Figures 1-5 [file 41419_2020_2258_MOESM1_ESM.pdf]
